# Diversity in Secondary Metabolites Including Mycotoxins from Strains of *Aspergillus* Section *Nigri* Isolated from Raw Cashew Nuts from Benin, West Africa

**DOI:** 10.1371/journal.pone.0164310

**Published:** 2016-10-21

**Authors:** Yendouban Lamboni, Kristian F. Nielsen, Anita R. Linnemann, Yüksel Gezgin, Kerstin Hell, Martinus J. R. Nout, Eddy J. Smid, Manuele Tamo, Martinus A. J. S. van Boekel, Jakob Blæsbjerg Hoof, Jens Christian Frisvad

**Affiliations:** 1 International Institute of Tropical Agriculture, Cotonou, Benin; 2 Department of Biotechnology and Bioengineering, Technical University of Denmark, Lyngby, Denmark; 3 Food Quality and Design Group, Wageningen University, Wageningen, The Netherlands; 4 Laboratory of Food Microbiology, Wageningen University, Wageningen, The Netherlands; 5 Department of Bioengineering, Ege University, Izmir, Turkey; Georg-August-Universitat Gottingen, GERMANY

## Abstract

In a previous study, raw cashew kernels were assayed for the fungal contamination focusing on strains belonging to the genus *Aspergillus* and on aflatoxins producers. These samples showed high contamination with *Aspergillus* section *Nigri* species and absence of aflatoxins. To investigate the diversity of secondary metabolites, including mycotoxins, the species of *A*. section *Nigri* may produce and thus threaten to contaminate the raw cashew kernels, 150 strains were isolated from cashew samples and assayed for their production of secondary metabolites using liquid chromatography high resolution mass spectrometry (LC-HRMS). Seven species of black Aspergilli were isolated based on morphological and chemical identification: *A*. *tubingensis* (44%), *A*. *niger* (32%), *A*. *brasiliensis* (10%), *A*. *carbonarius* (8.7%), *A*. *luchuensis* (2.7%), *A*. *aculeatus* (2%) and *A*. *aculeatinus* (0.7%). From these, 45 metabolites and their isomers were identified. Aurasperone and pyranonigrin A, produced by all species excluding *A*. *aculeatus* and *A*. *aculeatinus*, were most prevalent and were encountered in 146 (97.3%) and 145 (95.7%) isolates, respectively. Three mycotoxins groups were detected: fumonisins (B_2_ and B_4_) (2.7%) ochratoxin A (13.3%), and secalonic acids (2%), indicating that these mycotoxins could occur in raw cashew nuts. Thirty strains of black Aspergilli were randomly sampled for verification of species identity based on sequences of β-tubulin and calmodulin genes. Among them, 27 isolates were positive to the primers used and 11 were identified as *A*. *niger*, 7 as *A*. *tubingensis*, 6 as *A*. *carbonarius*, 2 as *A*. *luchuensis* and 1 as *A*. *welwitschiae* confirming the species names as based on morphology and chemical features. These strains clustered in 5 clades in *A*. section *Nigri*. Chemical profile clustering also showed also 5 groups confirming the species specific metabolites production.

## Introduction

*Aspergillus* section *Nigri* also known as black Aspergilli are among the most common fungi responsible for food spoilage and bio-deterioration of other materials [1], also causing substantial impact on food safety due to their mycotoxins production. They are known to produce the mycotoxins ochratoxin A [2], fumonisins B_2_, B_4_ and B_6_ [3,4] as well as numerous other compounds with poorly investigated activities [4,5]. On the other hand, black Aspergilli are also reported to be of biotechnological importance due to their use in the fermentation industry, for example in their ability to produce hydrolytic enzymes and organic acids [6]. *Aspergillus luchuensis* is reported to be extensively used in Asia for koji production [7]. Moreover, many *A*. *niger* processes have been classified as GRAS (Generally Recognized As Safe) by the Food and Drug Administration of the US government [1] despite the ability of *A*. *niger* to produce ochratoxin A and fumonisins. However these mycotoxins seem not to be produced under submerged conditions [8].

Black Aspergilli are one of the most complicated species complexes to classify and identify, and the taxonomy of strains in the *A*. section *Nigri* has been studied and debated for decades. In 1934, Mosseray described 35 species of black Aspergilli [9]. Later, that number was reduced to 12 species by Raper and Fennell [10]. In 1984, based on morphological features, Al-Musallam [11] revised the taxonomy of *niger* group to 7 species: *A*. *japonicus*, *A*. *carbonarius*, *A*. *ellipticus*, *A*. *helicothrix*, *A*. *heteromorphus*, *A*. *foetidus* and *A*. *niger*. While, in 2009, Nielsen et al. [4] reported 18 species in the black Aspergilli group with *A*. *niger*, *A*. *tubingensis*, *A*. *brasiliensis*, *A*. *acidus*, *A*. *carbonarius* and *A*. *ibericus* as the most common ones. In 2012, Jurjević et al. [12] added *A*. *floridensis* and *A*. *trinidadensis* as new species to the *A*. section *Nigri*. Recently, Varga et al. [13] revisiting the species in *A*. section *Nigri*, added 4 other new species and concluded that the black Aspergilli group includes 26 taxa. Therefore, a polyphasic taxonomic approach [14], has been used to accurately identify black Aspergilli at species level. These include morphological, physiological and biochemical characteristics of the isolates, e.g. using high performance liquid chromatography mass spectrometry (HPLC-MS) as well as DNA sequence analysis. The latter is presently based on the use of β-tubulin [15] and calmodulin [16] genes [7,13], as the ITS regions does not provide sufficient resolution [17].

Nuts are nutritious human foodstuffs [18] because of their high content of protein, carbohydrates, vitamins, essential minerals and especially unsaturated fatty acids. Nuts are consumed in both developing and developed countries by all age groups and across all social strata [19]. Among tree nuts, cashew nuts are known for their high minerals content (e.g. copper, iron and phosphate) and vitamins (e.g. thiamine, vitamin E and pyridoxine) [18]. In tropical regions of Africa, 48% of the world’s cashew nuts are produced, making them crops of high economic importance [20], and in 2011, cashew nut export contributed about 150 million US dollars to the gross domestic product of Benin [21] accounting for 8% of the national export revenues.

Since stored nuts generally have a low water activity, their spoilage association consists mainly of fungi and members of *A*. section *Nigri* have been reported to contaminate cashew nuts [22].

Although strains of *A*. section *Nigri* have been found on cashew nuts, very little is known about the risk of mycotoxins contamination from black Aspergilli on cashew kernels. Therefore, the objective of this study was to screen the mycotoxins and other metabolites that can be produced by *A*. section *Nigri* strains isolated from raw cashew kernels and, based on their metabolite production, to determine which species are prevalent on kernels from Benin. To accomplish these goals, isolated black Aspergilli were assayed for their mycotoxins and other secondary metabolites diversity by LC-HRMS on a LC-time-of-flight mass spectrometry instrument (LC-TOFMS) and representative strains were identified using molecular methods.

## Materials and Methods

### Chemicals and reagents

All solvents used for chemical analysis were LC-MS grade. Methanol, acetonitrile, 2-propanol, formic acid were LC-MS grade, while ethyl-acetate and dichloromethane were HPLC grade. All were purchased from Sigma Aldrich (Fluka Analytical, Denmark). Purified water was obtained by using a Milli-Q water purification system (Millipore Synergy® UV, Molsheim, France).

### Fungal isolates and growth conditions

One hundred and fifty strains belonging to *Aspergillus* section *Nigri* isolated from cashew nut samples from northern Benin were used in this study. These strains were obtained from Lamboni et al. [23]. Cashew nuts were sampled in the main cashew production area covering two agro-ecological zones lying within latitudes 8°1’ and 12°3’ N and longitudes 0°8’ and 3°8’ E, with an unimodal rainfall distribution averaging 900 mm to 1000 mm annually and maximum temperatures varying from 28°C to 40°C. Based on the agreement between the International Institute of Tropical Agriculture located in Benin and the Beninese Government, any other specific permissions were not required for sampling cashew nut within the study area. In total, 70 nuts samples were randomly selected in fourteen different locations.

After collection, the cashew shell was cut and the kernel (2 cotyledons) aseptically extracted, plated on dichloran 18% glycerol agar (DG18, Oxoid, Basingstoke, Hampshire, UK) [24] and incubated at 25°C in the dark for 7 days. Four cotyledons were plated per Petri dish, either in five replicates for surface sterilization (SS) (0.4% aqueous solution of sodium hypochlorite) or in two replicates for direct plating (DP), giving a total of 1960 cultured cotyledons. Both culturing methods were used to enable the growth of conidia present in the inner and the outer part of the cotyledons. According to taxonomic schemes and illustrations in Samson et al. [25], colonies belonging to *A*. section *Nigri* were first isolated on Czapek yeast autolysate agar (CYA) [24] and later 3 point inoculated on Yeast extract sucrose agar (YES) [24]. The plates were incubated at 25°C in the dark for 5 days. From the centre of fugal colonies, three 5-mm agar plugs were taken with an aseptic steel drill and pooled together into the same vial and stored at 4°C for further extraction.

### Plug extraction

A one step extraction method was used by adding 0.5 ml of a mixture of ethyl acetate-dichloromethane-methanol (3:2:1, v/v/v) with 1% (v/v) formic acid to the vials containing the agar plugs. The plugs were then extracted in an ultrasonic bath for 60 min. The supernatant was transferred to a new vial, evaporated to complete dryness using N_2_ flow, and re-dissolved in 500 μl of methanol assisted by ultrasonication for 20 min, and the aliquots filtered into an HPLC vial using a 0.45 μm polytetrafluoroethene (PTFE) filter.

### UHPLC-QTOF-MS analysis

Analyses were performed using ultra-high-performance liquid chromatography (UHPLC) with diode array detector and maXis 3G QTOF mass spectrometer (MS) (Bruker Daltonics, Bremen, Germany) equipped with an electrospray source (ESI) and connected to an Ultimate 3000 UHPLC system (Dionex, Sunnyvale, USA) equipped with a Kinetex 2.6-μm C_18_, 100 mm × 2.1 mm column (Phenomenex, Torrance, CA) [26]. A linear water-acetonitrile gradient was used (buffered with 20 mM formic acid) starting from 10% (v/v) acetonitrile and increased to 100% in 10 min, maintaining for 3 min before returning to the starting conditions. MS was performed in ESI^+^, the scan range m/z 100–1000, with a mass accuracy < 1.5 ppm [26]. UV/VIS spectra were collected at wavelengths from 200 to 700 nm. Data processing was performed using DataAnalysis 4.0 and Target Analysis 1.2 (Bruker Daltonics) by the aggressive dereplication approach [26], using a database of 495 known and putative compounds from black Aspergilli, tentatively identifying them based on accurate mass (deviation < 1.5 ppm) and isotopic pattern (isotope fit < 50) [26]. For saturated peaks (>10^6^ counts/sec) a manual verification of the accurate mass was made in the front and the tail of the peak. A further database of 1500 reference standards, tentatively identified compounds were also used along with a small 50 compounds database of peaks observed in sample blanks. All major peaks (observed in the BPC chromatograms) not tentatively identified by the approach were added to the search list as unknown compounds for mapping.

### DNA sequencing of *Aspergillus* section *Nigri* strains

#### *Aspergillus* isolates and growth conditions

Thirty strains of *Aspergillus* section *Nigri* isolated from raw cashew nuts were randomly selected for diagnostic PCR and sequencing. The strains were 3 point inoculated on separate Petri dishes containing Czapek yeast autolysate (CYA) agar and incubated in micro perforated plastic bags at 25°C for 7 days in the dark, to ensure extensive conidiation of the colonies. From these cultivations on solid media, we prepared stock suspensions for further inoculations and harvested conidia to make suspensions in 5 ml glass tubes containing autoclaved milli-Q water supplemented with 0.05% Tween 80. Conidia were inoculated at 3 points equidistant from the centre, on CYA and incubated in micro perforated plastic bags in the dark at 25°C for 3 days to favour mycelial growth and reduce the total conidiation as this would inhibit tissue-PCR. The Petri dishes were kept at 4°C for sampling.

#### Tissue-PCR for molecular identification of fungal isolates

Tissue PCR alleviates the need for genomic DNA extraction, as fungal mycelial tissue was the direct source for template DNA in PCR reactions amplifying partial genes encoding calmodulin and β-tubulin. PCR tubes containing a total volume of 40 μl had the following components mixed in milli-Q H_2_O; 1X Phire PCR buffer (ThermoFisher Scientific, USA), 200 μM dNTP mix (Invitrogen, Merelbeke, Belgium), 0.25 μM forward and reverse primers and 0.7 U Pfu X7 polymerase [27]. We based molecular identification of the thirty strains on the amplification of two partial genes encoding β-tubulin and calmodulin. The selected β-tubulin primers were T10-F-ACGATAGGTTCACCTCCAGAC [28], and Bt2b-R-ACCCTCAGTGTAGTGACCCTTGGC [15], and for calmodulin Cmd5-F CCGAGTACAAGGARGCCTTC and Cmd6-R CCGATRGAGGTCATRACGTGG [29]. A sterile pipette tip was used to streak 1 to 3 mm of peripheral mycelium in two replicates. Distribution of the fungal tissue on the pipette tip to PCR tubes resulted in two tubes with different amounts of biomass and thereby template DNA for PCR.

The amplification was performed in Agilent SureCycler 8800 Thermal Cycler (Agilent Technologies Inc., Santa Clara CA, USA). The amplification process consisted of an initial denaturation step of 30 min at 98°C to release template DNA from fungal debris, followed by 35 cycles of touch-down PCR with 10 s at 98°C (denaturation), 30 s at 61–52°C (primer annealing) and 1 min at 72°C (extension), and a final extension step of 5 min at 72°C. We verified purity of the amplification products by agarose gel electrophoresis in 1% TAE buffer (tris-acetate-EDTA (Ethylenediaminetetraacetic acid)) stained by SYBR Safe DNA Gel Stain (ThermoFisher Scientific, USA) and visualized by UV-light. If more than one product was observed after electrophoresis, PCR products were purified prior to DNA sequencing using the GFX PCR DNA and Gel Band Purification Kit (GE Healthcare UK limited, Buckinghamshire, UK). PCR products were sequenced by GATC Biotech (Constance, Germany).

#### Phylogenetic analysis of sequence data

The identity of the β-tubulin and calmodulin gene sequences was determined using Basic Local Alignment Search Tool for nucleotide (BLASTN) algorithm in the National Centre for Biotechnology Information (NCBI) GenBank database (http://blast.ncbi.nlm.nih.gov/Blast.cgi). They were then transformed into multi FASTA format using DNA Baser software. Phylogenetic analyses and molecular evolutionary were conducted using MEGA (Molecular Evolutionary Genetics Analysis) version 6.0 [30]. Sequences were pairwise aligned by Clustal W method [31] and trimmed both sides up to the same nucleotide position. Phylogenetic trees were prepared using the maximum likelihood method. Evolutionary distances were calculated by using the Jukes-Cantor [32] model embedded in the MEGA package. Bootstrap values were calculated from 1000 replications after complete deletion of all positions containing gaps or missing data. To compare with cluster output of DNA Baser, secondary metabolites of strains were grouped using MultiExperiment Viewer (MeV v4.2).

## Results

### Mycotoxins and other metabolites diversity from *Nigri* group on cashew nuts

The mycotoxins and other metabolites produced by strains of *Aspergillus* section *Nigri* on YES agar are presented in **Table 1**. From the 150 isolates used for metabolites profiling, 66 strains (44%) belonged to *A*. *tubingensis*, 48 strains (32%) to *A*. *niger* (with a chemical profile similar to *A*. *welwitschiae*), 15 strains (10%) to *A*. *brasiliensis*, 13 strains (8.7%) to *A*. *carbonarius*, 4 strains (2.7%) to *A*. *luchuensis* (synonyms to *A*. *kawachi* or *A*. *acidus*), 3 strains (2%) to *A*. *aculeatus* and 1 strain (0.7%) to *A*. *aculeatinus*.

**Table 1:** Mycotoxins and other secondary metabolites of *Aspergillus* section *Nigri* isolated from cashew nuts.

		Mycotoxins/metabolites^a^	R.T. (min)	n (pos)	*A*. *tubingensis*	*A*. *niger / welwitschiae*	*A*. *brasiliensis*	*A*. *carbonarius*	*A*. *luchuensis*	*A*. *aculeatus*	*A*. *aculeatinus*
		Total strains = 150			*66(7)*^b^	*48(11)*	*15(0)*	*13(7)*	*4(2)*	*3(2)*	*1(1)*
**1**	**A**	Fumonisin B_2_*	5.37	04	_	+ (8.3)^c^	_	_	_	_	_
**2**		Fumonisin B_4_*	5.78	04	_	+ (8.3)	_	_	_	_	_
**3**		Ochratoxin B	5.90	08	_	+ (10.4)	_	++ (23.1)	_	_	_
**4**		Ochratoxin A	6.63	20	_	+ (14.6)	_	++ (100)	_	_	_
**5**		Secalonic acids A, D, F	7.30	03	_	_	_	_	_	+ (100)	_
**6**		Nigragillin	1.56	129	+++ (95.5)^c^	++ (93.8)	+ (100)	_	+ (100)	_	_
**7**		Pyranonigrin A	2.27	145	+++ (97.0)	++ (100)	++ (100)	+ (100)	++ (100)	_	_
**8**	**B**	Nigerazine A, B	2.98	03	_	+ (4.2)	_	_	_	_	++ (100)
**9**		Nigerapyrone E	3.18	99	++ (42.4)	++ (87.5)	++ (80.0)	+ (100)	+ (50.0)	_	_
**10**		Tensyuic acid A / F	3.18	116	+ (51.5)	+++ (83.3)	+ (66.7)	+ (100)	+++ (100)	_	_
**11**		Pyranonigrin B / C	3.60	65	++ (37.9)	++ (33.3)	++ (53.3)	+ (100)	++ (75.0)	_	_
**12**		Pyranonigrin D	3.77	17	+ (1.5)	+ (2.1)	++ (6.7)	+++ (100)	+++ (25.0)	_	_
**13**		Fonsecin	4.30	144	+++ (97.0)	++ (100)	++ (100)	+++ (100)	+ (100)	_	_
**14**		Tensidol A	4.42	72	+ (31.8)	++ (75.0)	+ (93.3)	_	_	_	_
**15**		Pyrophen	4.45	79	+ (28.8)	+++ (91.7)	++ (93.3)	_	+ (50.0)	_	_
**16**		Atromentin	4.52	145	+ (100)	+ (100)	+ (100)	+ (100)	+ (100)	_	_
**17**		Tensyuic acid B	4.52	67	+ (31.8)	+ (41.7)	+ (66.7)	++ (100)	+ (25.0)	_	_
**18**		Funalenone	5.08	137	++ (100)	++ (97.9)	++ (93.3)	+ (46.2)	++ (100)	_	_
**19**		Rubrofusarin	5.27	142	++ (95.5)	+++ (97.9)	++ (100)	+++ (100)	++ (100)	_	+ (100)
**20**		Orlandin	5.32	07	_	+ (14.6)	_	_	_	_	_
**21**		Asperazine	5.35	70	+++ (100)	_	_	_	+ (100)	_	_
**22**		Tensyuic acid C or D	5.41	39	+ (7.6)	+ (25.0)	+ (20.0)	++ (100)	+ (25.0)	_	_
**23**		Nigerasperone A	5.42	57	+ (28.8)	++ (58.3)	++ (60.0)	_	_	_	_
**24**		Tensidol B (Pestalamide A)	5.42	70	++ (31.8)	++ (68.8)	+ (86.7)	_	_	_	_
**25**		Fonsecin B	5.58	140	++ (97.0)	++ (93.8)	+ (100)	+++ (100)	+ (100)	_	_
**26**		Malformin A_2_	5.69	102	+++ (83.3)	+ (72.9)	+ (93.3)	_	_	_	_
**27**		Tubingensin A or B	5.90	03	+ (3.0)	_	_	_	+ (25.0)	_	_
**28**		Malformin C	6.12	116	+++ (93.9)	++ (81.3)	+ (100)	_	+ (50.0)	_	_
**29**		Kotanin	6.57	06	_	+ (12.5)	_	_	_	_	_
**30**		Nominine	10.1	08	++ (7.6)	_	_	_	+ (25.0)	++ (66.7)	_
**31**		Antafumicin A or B	3.56	04	_	_	_	_	+ (100)^c^	_	_
**32**		Aurasperone C	5.95	146	+++ (100)	++ (100)	+ (100)	++ (100)	++ (100)	_	_
**33**		Aurasperone F	6.29	145	+++(100)	+++ (100)	++ (100)	++ (100)	+ (75.0)	_	_
**34**		Aurasperone E	6.58	133	+ (87.9)	++ (95.8)	++ (100)	+ (100)	++ (75.0)	_	_
**35**		Aurasperone B	6.61	141	+ (97.0)	++ (95.8)	++ (100)	++ (100)	++ (75.0)	_	_
**36**		Flavasperone	7.03	137	+++ (90.9)	+++ (95.8)	+++ (100)	++ (100)	+ (50.0)	_	_
**37**		Nafuredin	9.22	55	++ (33.3)	++ (56.3)	+ (20)	_	+ (75.0)	_	_

* Small peak of fumonisin B_6_ were also detected when B_4_ and B_2_ was detected.

^a^ Divided in 2 groups: A = mycotoxins known as toxic compounds for humans, B = other secondary metabolites.

^b^ Number of strains of each species, with in brackets, number of strains selected for DNA sequencing.

(+) Detected with relative abundance within a line (+++ high production, ++ average production and + low production); (-) not detected; n (pos) = total number of positive strains for a given mycotoxin/metabolite; R. T. = retention time in min using ultra-high-performance liquid chromatography quadrupole time of flight mass spectrometry. Positive electrospray ionization (*ESI*^+^, *m/z* 100–1000).

^c^ In bracket is the percentage of strains that produced a given mycotoxin/metabolite.

In total, 45 metabolites including their isomers were identified during UHPLC-QTOF-MS analysis within retention times (RT) ranging from 1.56 min (nigragillin) to 10.1 min (aflavinine) (**S1 Table**). Aurasperone C (positive in 97.3%), aurasperone F (96.7%), pyranonigrin A (96.7%), and fonsecin (96%) were the metabolites identified in most of the strains of *A*. section *Nigri*. The metabolites that were rarely produced by strains of *A*. section *Nigri* were secalonic acids (2%), tubingensins (2%), antafumicins (2.7%), fumonisins (2.7%), kotanin (4%) and ochratoxin A (5.3%). The detection of orlandin, kotanin and fumonisin B_2_, B_4,_ B_6_ was specific for *A*. *niger* whereas the presence of antafumicin A and B was specific for *A*. *luchuensis*. Secalonic acids were specific for *A*. *aculeatus*.

Secalonic acids, atromentin, asperazine and aurasperone C were produced consistently by all the strains in a species. The mycotoxin fumonisin B_2_ (2.7%) was detected in strains belonging to *A*. *niger* whereas ochratoxin A (13%) and ochratoxin B (5.3%) were produced by strains of both *A*. *niger* and *A*. *carbonarius*.

For example, in **Table 1**with UHPLC-QTOF MS, nigragillin was produced by 129 (86%) of the 150 strains studied. Nigragilin was produced by 95% of the strains of *A*. *tubingensis* (64/66), 94% of *A*. *niger* (46/48) and 100% of both the strains of *A*. *brasiliensis* (15) and *A*. *luchuensis* (4).

**Fig 1:**
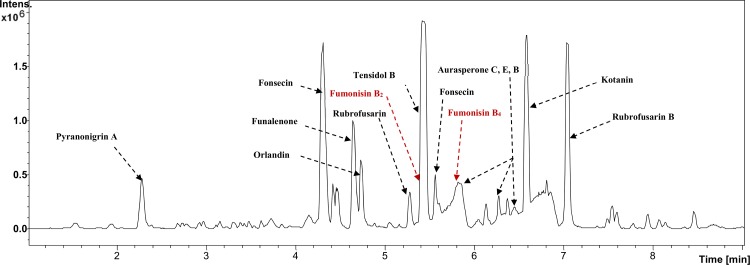
Base Peak Chromatogram (BPC) of *Aspergillus niger* extract. The Analysis was done by reversed phase ultra-high-performance liquid chromatography quadrupole time of flight mass spectrometry. Positive electrospray ionization (*ESI*^+^, *m/z* 100–1000). *A*. *niger* was cultured on yeast extract sucrose agar for 5 days in dark. The BPC showed the production of fumonisin B_2_ and fumonisin B_4_ and other secondary metabolites.

An example of Base Peak Chromatogram (BPC) of *A*. *niger* extract is shown in **Fig 1**where several compounds were identified including fumonisin B_2_ and fumonisin B_4_.

### Phylogenetic analysis

We examined the genetic relatedness of 30 randomly taken strains of *Aspergillus* section *Nigri* using nucleotide sequences of β-tubulin and calmodulin genes. **Table 2**summarizes the species names based on their metabolite production which was confirmed by sequencing data. Eleven isolates were identified as *A*. *niger*, 7 as *A*. *tubingensis*, 6 as *A*. *carbonarius*, 2 as *A*. *luchuensis*, 2 as *A*. *aculeatus*, 1 as *A*. *aculeatinus* and 1 as *A*. *welwitschiae*. Three of the isolates (2 of *A*. *aculeatus* and 1 of *A*. *aculeatinus*) did not match with the β-tubulin and calmodulin genes used.

**Table 2:** *Aspergillus* section *Nigri* strains isolated from cashew nuts characterized by sequence analysis.

	Samples Id	Sequencing Id	method ^a^	Location ^b^	Species ^c^	GenBank accession number ^d^
1	8714	Lyl1	SS	Penessoulou	*A*. *tubingensis*	KX769852
2	8715	-	SS	Penessoulou	***A*. *aculeatus***	***-***
3	8716	-	SS	Penessoulou	***A*. *aculeatus***	***-***
4	8726	-	SS	Kolokonde	***A*. *aculeatinus***	***-***
5	8726	Lyl2	SS	Kolokonde	*A*. *niger*	KX769853
6	8741	Lyl3	SS	Patargo	*A*. *niger*	KX769854
7	8743	Lyl42	SS	Patargo	*A*. *carbonarius*	KX769875
8	8749	Lyl33	SS	Pira	*A*. *niger*	KX769866
9	8755	Lyl4	SS	Nagayile	*A*. *niger*	KX769855
10	8764	Lyl5	SS	Birni	*A*. *niger*	KX769856
11	8765	Lyl6	SS	Birni	*A*. *luchuensis*	KX769857
12	8775	Lyl43	SS	Alafiarou	*A*. *carbonarius*	KX769876
13	8710	Lyl7	DP	Penessoulou	*A*. *welwitschiae*	KX769858
14	8718	Lyl8	DP	Kolokonde	*A*. *niger*	KX769859
15	8735	Lyl9	DP	Chabikouma	*A*. *niger*	KX769860
16	8739	Lyl45	DP	Patargo	*A*. *carbonarius*	KX769878
17	8741	Lyl11	DP	Patargo	*A*. *luchuensis*	KX769861
18	8749	Lyl12	DP	Pira	*A*. *niger*	KX769862
19	8761	Lyl13	DP	Nagayile	*A*. *tubingensis*	KX769863
20	8768	Lyl14	DP	Birni	*A*. *niger*	KX769864
21	8809	Lyl44	DP	Tchaourou	*A*. *carbonarius*	KX769877
22	8714	Lyl15	DP	Penessoulou	*A*. *carbonarius*	KX769865
23	8799	Lyl34	DP	Kilibo	*A*. *tubingensis*	KX769867
24	8792	Lyl35	SS	Toui	*A*. *tubingensis*	KX769868
25	8800	Lyl36	SS	Kilibo	*A*. *tubingensis*	KX769869
26	8714	Lyl37	SS	Penessoulou	*A*. *tubingensis*	KX769870
27	8718	Lyl38	SS	Kolokonde	*A*. *tubingensis*	KX769871
28	8739	Lyl39	SS	Patargo	*A*. *niger*	KX769872
29	8758	Lyl40	SS	Nagayile	*A*. *niger*	KX769873
30	8801	Lyl41	SS	Kilibo	*A*. *carbonarius*	KX769874

^a^ SS = strains isolated after surface sterilization of cashew kernels; DP = strains isolated after direct plating of cashew kernels. In both methods, cashew kernels were first plated on Dichloran 18% glycerol agar (DG18, Oxoid). Strains belong to *A*. section *Nigri* were isolated on Czapek yeast autolysate agar (CYA) and later inoculated on Yeast extract sucrose agar (YES). The plates were incubated at 25°C in the dark for 5 days.

^b^ All locations are in northern Benin within latitudes 8°1’ and 12°3’ N and longitudes 0°8’ and 3°8’ E, with an unimodal rainfall distribution of 900 mm to 1000 mm annually and maximum temperatures varying from 28°C to 40°C.

^c^ No PCR products was obtained for the partial β-tubulin and calmodulin gene sequences for the species in bold. Their identification relied only on morphology and their metabolite profile.

^d^ GenBank accession numbers based on partial calmodulin genes used during PCR amplification.

**Fig 2:**
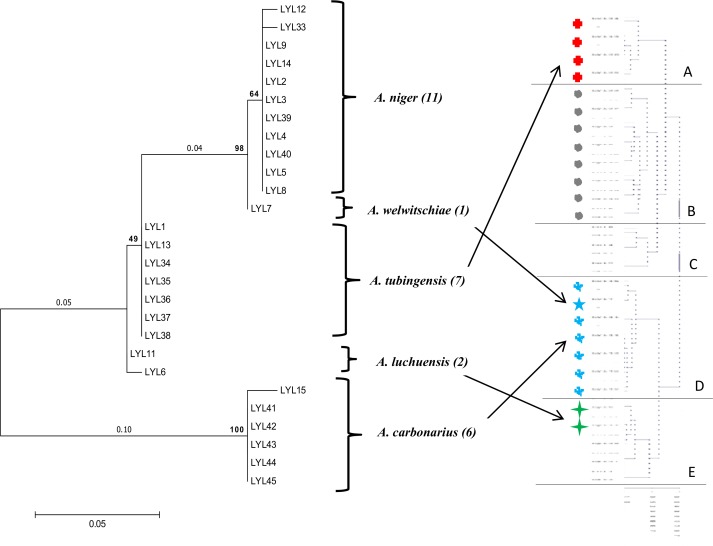
Phylogenetic trees based on combined sequences data of β-tubulin and calmodulin of 27 strains of *Aspergillus* section *Nigri*, with clustering based on metabolites profile. Phylogenetic analyses and molecular evolutionary were conducted using MEGA version 6.0. The identity of the gene sequences was determined using BLASTN algorithm. The alignment was performed using the Clustal W program. Nucleotide divergences were estimated according to the Jukes-Cantor model. The numbers above branches are bootstrap values. The evolutionary history was inferred using the maximum likelihood method. The chemical clustering was performed using MultiExperiment Viewer (MeV v4.2).

The phylogenetic relationship between the isolates of *A*. section *Nigri* species was illustrated in the maximum likelihood method analysis. Twenty seven isolates matched either with β-tubulin and/or calmodulin genes during PCR amplification. The 27 sequences were aligned and resulted in the formation of 5 clades (**Fig 2**). All the *A*. *niger* strains (11) clustered together, as did the *A*. *tubingensis* strains (7) and *A*. *carbonarius* strains (6). *A*. *welwitschiae* strain was separated from *A*. *niger* sequences with 98% bootstrap value. Two strains of *A*. *luchuensis* clustered to form a clade.

The hierarchical clustering based on chemical compounds of the 27 strains, showed 5 major clusters similar to the genetic clades (**Fig 2**). Some of the strains in *A*. *tubingensis* group clustered (A) whereas the others clustered together with strains of the *A*. *luchuensis* clade (E). Strains of *A*. *niger* clustered in 2 groups (B and C) whereas *A*. *welwitschiae* joined strains of *A*. *carbonarius* to form cluster D (**Fig 2**). The hierarchical clustering revealed aflavinin, tubingensin, orlandin, secalonic acids, fumonisins and ochratoxins as key compounds for chemical clustering (**S1 Fig**).

## Discussion

One hundred and fifty strains of *Aspergillus* section *Nigri* were used for mycotoxin and other secondary metabolite profiling using ultra-high-performance liquid chromatography. All the 45 chemical compounds identified pertained to black Aspergilli as previously described by Nielsen et al. [4]. Some of these natural products are known to be toxic to human and animals. These where classified in **Table 1**as group A and included fumonisins and ochratoxin A, which were reported for *Aspergillus niger* previously[33]. According to Mogensen et al. [34] and Noonim et al. [35], up to 75% of *A*. *niger* isolates produce fumonisins and 41% produce ochratoxins. Also, Massi et al. [36] reported 74% of *A*. *niger* to be fumonisin B_2_ producers while 32% were ochratoxin A producers. In our results, among the 48 strains of *A*. *niger* isolated, only 9% (4 strains) produced fumonisins and 15% (7 strains) produced ochratoxins. The difference could be due to the type of commodities from which the *A*. *niger* strains were isolated. The effect of the food matrix was demonstrated by Vaamonde et al. [37] who studied the variability of aflatoxin production by strains of *A*. section *Flavi* in peanut, wheat and soybean. The isolates of Mogensen et al. [34] were from raisins and those from Noonim et al. [35] from dried coffee bean samples. The food matrix of raisins (16% of moisture, 25 to 30% of sugars and ˂1% of lipids) is different from that of dried green coffee (12–13% of moisture, ˂1% of sugars and 4 to15% of lipids) and much more different from that of cashew nuts (8 to 9% of moisture, 1 to 8% of sugars and 60 to 64% of lipids). Isolates from Massi et al. [36] were from different food commodities: dried fruits, Brazil nuts, coffee beans, grapes cocoa and onions. In addition, cashew nuts also have a thick shell that constitutes a first barrier to microbial contamination [38]. It is known that cashew shells contain tannins that are able to suppress mycotoxin formation [39] and probably alter the gene expression by the fungi. More so, on Pistachio nuts, Marin et al. [40] noticed only 5% of *A*. *niger* to be ochratoxin A positive.

The geographic origin of a strain can reportedly influence its mycotoxin production. Isolates of *A*. *flavus* from various geographic regions have revealed differences in the proportions of isolates that produce low, medium and high amount of aflatoxins [41]. This could also apply to fumonisin and ochratoxin production by black Aspergilli. Samson et al. [42] reported ochratoxin production from species of *A*. section *Nigri* isolated from different food matrices collected from various regions. Moreover, Perrone et al. [43] reported that 33% of *A*. *niger* isolated from grapes in Italy produced ochratoxin A. In our study we did not notice simultaneous production of both fumonisins and ochratoxins from the same strains of *A*. *niger*, where Frisvad et al. [8] reported that up to 10% of *A*. *niger* strains may produce both mycotoxins. Ochratoxin A production rate can be overestimated in some studies as HPLC with fluorescence detection (even using immunoaffinity purification), can provide false positives [44] which unfortunately has been extensively reported for *A*. *tubingensis*. [43,45]. Ochratoxin A production from *A*. *tubingensis* was not detected during our screening process, confirming the report of Nielsen et al. [4] and Storari et al. [44] concerning this. Also, in accordance with Frisvad et al. [8], strains of *A*. *tubingensis*, *A*. *brasiliensis* and *A*. *luchuensis* did not produce fumonisins or ochratoxins.

*A*. *carbonarius* isolates always produced ochratoxin A as reported by several authors [4,46,47]. Our result was in accordance with this consistent production since all the 13 isolates of *A*. *carbonarius* showed ochratoxin A production.

Secalonic acids, reported as toxic metabolites of *A*. *aculeatus* [48] were noticed during our analysis, confirming their production by *A*. *aculeatus* as mentioned by Parenicová et al. [49]. These toxic compounds were not produced by *A*. *aculeatinus* from cashew nuts which is in contrast to the report by Noonim et al. [50].

Some secondary metabolites detected during our analysis, such as Aurasperones, Nigragillin, Malformins and Nigerazine and grouped as B in Table 1 are reported to be toxic compounds to plants, bacteria, and mice [5,51]. Asperazine was reported to have significant *in vitro* cytotoxicity against human leukemia [52] but no *in vivo* including bioavailability studies confirmed this. Malformins are currently being investigated for anti-cancer drug potential [53]. Altogether there are very few studies on other effects than *in-vitro* or in older studies intraperitoneal injection of compounds. However these do not include degradation in the body nor bioavailability of the compounds, and with a definition of mycotoxin being toxic through a natural route of exposure, such studies can only be considered indicative, but also highlights the need for testing these compounds in relevant animal models under relevant exposure conditions. Similar problems are reported in the *Aspergillus glaucus* group (and formerly *Eurotium*) [54,55]

DNA sequencing using β-tubulin and calmodulin genes was performed to validate morphology and extrolite profile based on identification of our isolates and their association to *A*. section *Nigri*. The use of a polyphasic approach, to identify and validate to species level isolates of fungi, was described by Frisvad [56], Oliveri et al. [14]. Our DNA sequences confirmed the species name identified by morphological and chemical characteristics and the phylogenetic tree shows that the main clades belong to the black Aspergilli. Perrone et al. [43] in a cluster analysis of 94 isolates of *A*. section *Nigri* identified the same clades confirming the fact that *A*. *tubingensis* and *A*. *niger* are the main clades of *A*. section *Nigri* as reported by Nielsen et al. [4]. Moreover, Samson et al. [57] confirmed the presence of these 4 different clades in *A*. section *Nigri* and grouped them as biseriate group of *Aspergillus* section *Nigri* in contrast to uniseriate group of *Aspergillus* including *A*. *aculeatus*.

The cluster analysis of the 27 strains using their metabolite profiles was similar to the clustering based on sequencing data. The secondary metabolites have been previously used most often in species recognition due to their high species specificity [58]. Samson et al. [57] mentioned that isolates of *Aspergillus* species usually produce a diverse range of secondary metabolites that are characteristic of the different groups of section of *Aspergillus*. They also reported that the production of a particular secondary metabolite is an efficient identification aid for allocating a species to section while profiles of secondary metabolites can be very effective in identifying an *Aspergillus* isolates to species. With few exceptions, this was effective during our analysis where the combined production of orlandin, fumonisins and kotanin was specific to *A*. *niger*, and the production of antafumicin A and B was specific to *A*. *luchuensis*.

## Conclusion

The diversity in secondary metabolites including mycotoxins from isolates of *Aspergillus* section *Nigri*, analysed using UHPLC-QTOF-MS, revealed several metabolites produced by 7 different species that contaminated cashew nuts samples from Benin. In pure cultures on a laboratory medium, ochratoxin A and fumonisins, the 2 main toxic compounds from black Aspergilli, were produced by strains of 2 predominant species in *A*. section *Nigri*, namely *A*. *niger* / *A*. *welwitchiae* and *A*. *carbonarius*, although *A*. *carbonarius* is unable to produce fumonisins. Ochratoxins and fumonisins were produced by a relatively little proportion of the isolates of *A*. *niger* and *A*. *carbonarius*, but it is well know that species of *A*. section *Nigri* are the most isolated on cashew kernels, given a substantial number of species that may produce mycotoxins in cashew nuts. Even though the presence of fungi has not always meant the presence of mycotoxins, the production of ochratoxin A and fumonisins by isolates on *A*. section *Nigri* on cashew nuts could constitute an additional and hidden problem in term of mycotoxins content, and can negatively affect cashew nut safety and the nutritional quality of the nuts.

There are no regulations on ochratoxin A and fumonisins for raw and processed cashew nuts like those of EU and WHO on aflatoxins. Nevertheless, these findings suggest more investigations in order to detect the presence and the levels of ochratoxin A and fumonisins and to evaluate their exact contribution to the total level of mycotoxins in cashew kernels. But immediate actions should emphasize on the prevention by strengthening post-harvest practices that can lower fungal contamination along the cashew nut value chain, mainly during nut storage, where high contamination of species belonging to black Aspergilli are noticed.

## Supporting Information

S1 Fig Hierarchical clustering based on metabolites profile of strains of *Aspergillus* section *Nigri*. NB: The chemical clustering was performed using MultiExperiment Viewer (MeV v4.2); In brackets (“”) are the chemicals with high probability of being species specific metabolites.
(DOCX)

S1 Table Metabolites profile of *Aspergillus* section *Nigri* isolated from cashew nuts from Benin. (XLSX)
