# Supplementary material for: Diversity in Secondary Metabolites Including Mycotoxins from Strains of Aspergillus Section Nigri Isolated from Raw Cashew Nuts from Benin, West Africa
Source: PLoS One. 2016 Oct 21;11(10):e0164310. doi: 10.1371/journal.pone.0164310 (PMC5074577; doi:10.1371/journal.pone.0164310)
Supplement: S1 Fig — NB: The chemical clustering was performed using MultiExperiment Viewer (MeV v4.2); In brackets (“”) are the chemicals with high probability of being species specific metabolites. (DOCX) [file pone.0164310.s001.docx]

**S1 Fig. Hierarchical clustering based on metabolites profile of strains of *Aspergillus* section *Nigri.***

NB: The chemical clustering was performed using MultiExperiment Viewer (MeV v4.2);

In brackets (“ ”) are the chemicals with high probability of being species specific metabolites.

A, *tubingensis* (LYL34, LYL13, LYL36, LYL35, LYL38, LYL37, LYL1)

A. *niger* (LYL33, LYL 39, LYL3, LYL9, LYL12, LYL14, LYL40, LYL5, LYL8, LYL4, LYL2)

A. *carbonarius* (LYL15, LYL41, LYL44, LYL43, LYL42, LYL45)

A. *welwitschiae* (LYL7)

A. *luchensis* ( LYL6, LYL11)
